# Hyaluromycin, a Novel Hyaluronidase Inhibitor, Attenuates Pancreatic Cancer Cell Migration and Proliferation

**DOI:** 10.1155/2016/9063087

**Published:** 2016-12-20

**Authors:** Shiro Kohi, Norihiro Sato, Atsuhiro Koga, Keiji Hirata, Enjuro Harunari, Yasuhiro Igarashi

**Affiliations:** ^1^Department of Surgery 1, University of Occupational and Environmental Health, Kitakyushu, Japan; ^2^Biotechnology Research Center and Department of Biotechnology, Toyama Prefectural University, Toyama, Japan

## Abstract

Pancreatic ductal adenocarcinoma (PDAC) is characterized by accelerated production and degradation of hyaluronan (HA), a major component of extracellular matrix involved in the malignant phenotype of cancer. In particular, increased hyaluronidase (HYAL) activity plays a critical role in cancer progression, at least in part, by producing low-molecular-weight- (LMW-) HA or small fragments of HA, suggesting HYAL as a target for cancer treatment. Hyaluromycin, a new member of the rubromycin family of antibiotics, was isolated from the culture extract of a marine-derived* Streptomyces hyaluromycini* as a HYAL inhibitor. We investigated the antitumor effects of hyaluromycin in PDAC cells. We examined the effects of hyaluromycin on the proliferation and migration of PDAC cells. To elucidate the mechanisms underlying the effect of hyaluromycin on PDAC cells, we examined the concentration of LMW-HA in the conditioned media after treating PDAC cells with hyaluromycin. We demonstrate that hyaluromycin inhibits proliferation and migration of PDAC cells. We also found that these antitumor effects of hyaluromycin were associated with a decreased concentration of LMW-HA and a decreased phosphorylation of ribosomal protein S6. Our results suggest that hyaluromycin is a promising new drug against this highly aggressive neoplasm.

## 1. Introduction

During cancer progression, hyaluronan (HA), a major component of extracellular matrix, plays a critical role in a variety of cellular processes, including proliferation, adhesion, migration, invasion, metastasis, and drug resistance. This is particularly true in pancreatic ductal adenocarcinoma (PDAC) which is almost universally characterized by a dense “desmoplastic” stroma enriched with HA. In addition to abnormal production of HA, accelerated processing (notably, degradation) of HA is central to the aggressive behaviors of cancer cells.

HA is degraded by specific enzymes called hyaluronidases (HYALs). In previous studies HYAL levels have been shown to be elevated in various cancers [1–3]. Furthermore, higher HYALs expression exhibits significantly higher invasion ability than lower HYALs expression in breast cancer cells [4]. We reported that HYAL1 is overexpressed in PDAC cell lines and tissues and that inhibition of HYAL activity significantly inhibits the migration of PDAC cells [5]. HYAL2 initially cleaves high-molecular-weight- (HMW-) HA into ~20 kDa fragments, which are further digested into smaller fragments by HYAL1 [6, 7]. Interestingly, low-molecular-weight- (LMW-) HA or small HA fragments, rather than HMW-HA, have been suggested to be essential for cancer progression in terms of invasion and metastasis [8]. We also reported LMW-HA stimulated PDAC cell migration [9]. Accumulating evidence suggests that HYAL has a critical role in tumor progression. Therefore, targeting HYAL could be a potential approach for cancer therapy.

In previous studies some HYAL inhibitors such as glycyrrhizin, sulfated hyaluronic acid (sHA), and dextran sulfate have been tested for antitumor activity in cancer cells [8, 10, 11]. However, there were no previous reports testing the effects of HYAL inhibitors on PDAC. Hyaluromycin, a new member of the rubromycin family of antibiotics, was isolated from the culture extract of a marine-derived* Streptomyces hyaluromycini* as a HYAL inhibitor [12]. Importantly, hyaluromycin has a 25-fold more potent inhibitory activity against HYALs than glycyrrhizin, a well-known HYAL inhibitor used in clinical settings [12]. In this study, we investigated the antitumor effects of hyaluromycin in PDAC cells. We also examined a possible molecular mechanism underlying the effects of hyaluromycin on PDAC cell behaviors.

## 2. Materials and Methods

### 2.1. Cell Lines and Reagent

We used PDAC cell lines, BxPC-3 and CFPAC-1 (American Type Culture Collection, Manassas, VA, USA). PDAC cell lines were maintained in RPMI1640 medium (Life Technologies, Grand Island, NY, USA) supplemented with 10% fetal bovine serum (FBS) (Life Technologies) and 1% streptomycin and penicillin (Life Technologies) in a 5% CO_2_ incubator at 37°C. Isolation of hyaluromycin was described previously [12]. Hyaluromycin was dissolved in DMSO, and we added equal volume of DMSO in control.

### 2.2. Cell Proliferation Assay

PDAC cells were plated at 1 × 10^5^ cells/well in growth media with or without various concentration of hyaluromycin and incubated for 1, 3, and 5 days. Then, cells were trypsinized and counted following trypan blue staining.

### 2.3. Cell Migration Assay

The migratory ability of cells was determined by transwell cell migration assay using cell culture inserts equipped with a filter membrane containing 8 *μ*m pores (BD Biosciences, Franklin Lakes, NJ). The lower chamber was filled with RPMI1640 containing 10% FBS. The upper chamber was filled with 4 × 10^4^ PDAC cells suspended in the RPMI1640 containing 1% FBS. Hyaluromycin was added to the upper and lower chambers in the beginning of migration assay. After 24 h incubation, the cells remaining on the upper side of the filters were removed. The cells on the bottom surface of the membrane were stained with hematoxylin and eosin and the number of cells that had migrated to the bottom surface of the membrane was counted in five randomly selected microscopic fields in each samples.

### 2.4. Measurements of LMW-HA Concentrations

The cells (1.0 × 10^5^ cells/mL) were cultured in a serum-free medium (RPMI1640 without FBS) for 24 hours and the culture medium was collected for measurements of LMW-HA concentrations. The culture medium sample was centrifuged at 14000 *g* for 10 minutes through Amicon Ultra-0.5 Centrifugal Filter Devices (MilliporeSigma, Darmstadt, Hessian, Germany) with a 100 kDa cutoff and the LMW-HA (<100 kDa) were collected [8, 13]. The concentration of LMW-HA was measured using Quantikine ELISA Hyaluronan Immunoassay (R&D Systems Inc., Minneapolis, MN, USA). Assays were triplicated and the average concentrations were determined.

### 2.5. Intracellular Signaling Pathway Assay

To elucidate the underlying mechanisms regulating the effect of hyaluromycin on PDAC cells, we examined the simultaneous detection of 18 important and well-characterized signaling molecules when phosphorylated or cleaved in PDAC cells by PathScan Intracellular Signaling Array Kit (Cell Signaling Technology, Trask lane, Danvers MA, USA). Cells were treated with or without hyaluromycin at 50 *μ*M. After 24 hours' treatment, cells were harvested and examined PathScan Intracellular Signaling Array Kit according to the manufacture's instruction.

### 2.6. Statistical Analysis

Statistical analyses were performed using SPSS statistical software version 21.0 (SPSS, Chicago, Illinois, USA). Student's *t*-test and Mann–Whitney *U* were used for group comparison. A *P* value of < 0.05 was considered statistically significant.

## 3. Results

**Figure 1:**
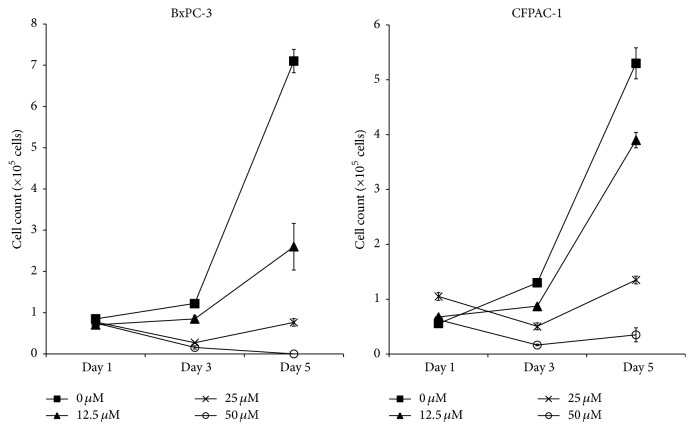
Hyaluromycin inhibited the growth of PDAC cell lines. Hyaluromycin did not affect the cell growth on day 1. However, hyaluromycin inhibited the cell growth on day 3 and day 5 in a dose-dependent manner.

First, we investigated the effects of hyaluromycin on the proliferation of PDAC cells. Two PDAC cell lines, BxPC-3 and CFPAC-1, were treated with various concentrations of hyaluromycin. As shown in Figure 1, hyaluromycin inhibited the growth of both PDAC cell lines in a dose dependent manner. Although the antiproliferative effect of hyaluromycin was modest at a concentration of 12.5 *μ*M, treatment at 25 *μ*M almost completely inhibited the cell proliferation for up to 5 days after treatment. Furthermore, treatment with 50 *μ*M of hyaluromycin resulted in a robust decrease in the number of cells, suggesting the induction of cell death.

**Figure 2:**
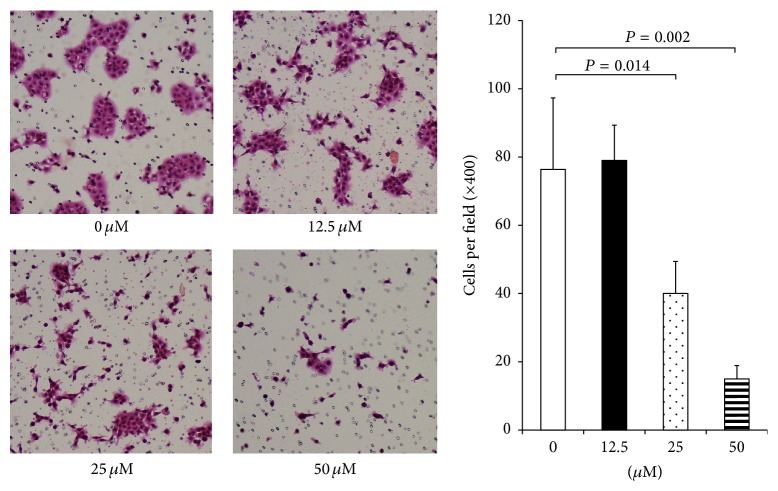
Hyaluromycin inhibited the migration of PDAC cell lines. After treatment with various concentrations (0–50 *μ*M) of hyaluromycin, the migrated cells were counted on day 1. Hyaluromycin significantly inhibited the migration of BxPC-3 at 25 and 50 *μ*M (25 *μ*M: *P* = 0.014, 50 *μ*M: *P* = 0.002).

We then determined the effects of hyaluromycin on the migration of PDAC cells using the transwell cell migration assay. After treatment with various concentrations (0–50 *μ*M) of hyaluromycin, the migrated cells were counted on day 1 (24 hours) when no significant effects on the cell proliferation were seen at these concentrations. Hyaluromycin significantly inhibited the migration of BxPC-3 at 25 and 50 *μ*M (Figure 2).

**Figure 3:**
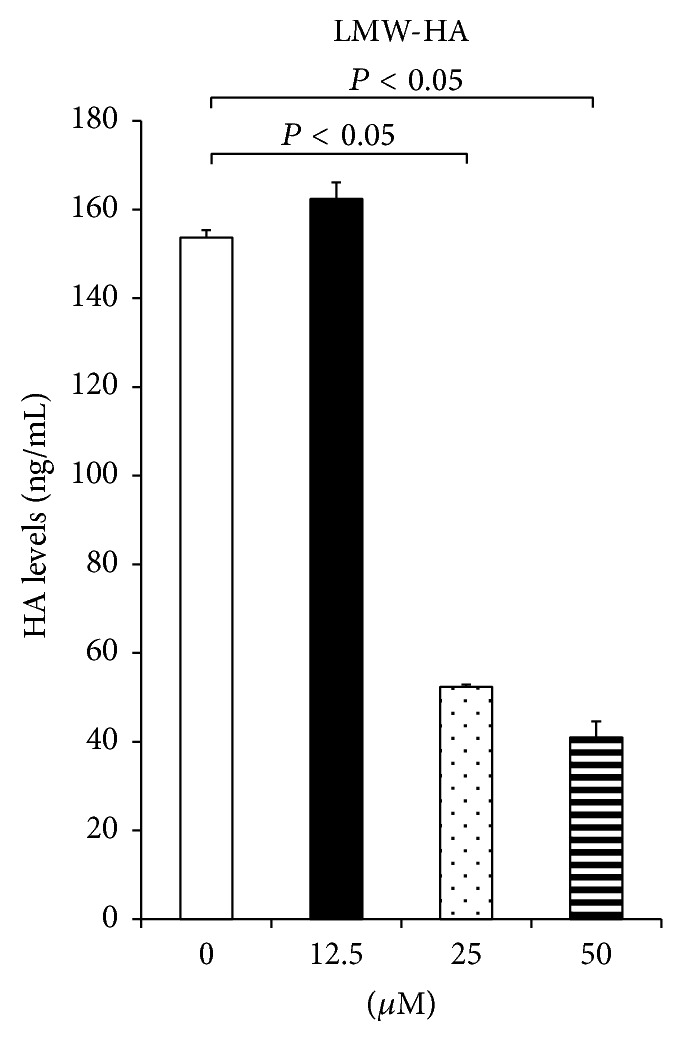
Hyaluromycin decreased the LMW-HA concentration in the conditioned media of BxPC-3. As compared to the untreated control, the concentrations of LMW-HA were significantly lower in the conditioned media after treatment with hyaluromycin at concentrations of 25 and 50 *μ*M (*P* < 0.05).

In an attempt to gain an insight into these antitumor properties of hyaluromycin, we measured the concentration of LMW-HA (<100 kDa) in the conditioned media after treating BxPC-3 with various concentrations (0–50 *μ*M) of hyaluromycin. As compared to the untreated control, the concentrations of LMW-HA were significantly lower in the conditioned media after treatment with hyaluromycin at concentrations of 25 *μ*M and 50 *μ*M (*P* < 0.05 for both concentrations; Figure 3).

**Figure 4:**
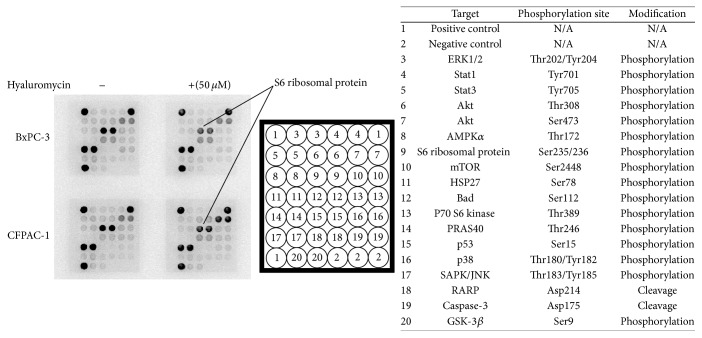
Hyaluromycin inhibited phosphorylation of ribosomal protein S6. We examined the simultaneous detection of 18 important and well-characterized signaling molecules when phosphorylated or cleaved in PDAC cells by PathScan® Intracellular Signaling Array Kit. Treatment with hyaluromycin at 50 *μ*M for 24 hours resulted in decreased phosphorylation (at Ser235/236) of ribosomal protein S6 in PDAC cell lines.

Finally, we thought to determine signaling pathways involved in the observed effects of hyaluromycin on PDAC cells. We used PathScan Intracellular Signaling Array Kit (Cell Signaling Technology, Trask lane, Danvers, MA, USA) which detects 18 important and well-characterized signaling molecules when phosphorylated or cleaved. Treatment with hyaluromycin at 50 *μ*M for 24 hours resulted in decreased phosphorylation (at Ser235/236) of ribosomal protein S6 in both cell lines (Figure 4).

## 4. Discussion

In the present study, we demonstrate that hyaluromycin, a novel HYAL inhibitor isolated from marine-derived* Streptomyces hyaluromycini*, inhibits the proliferation and migration of PDAC cells. We also found that these anticancer properties of hyaluromycin were associated with a decreased level of LMW-HA and decreased phosphorylation of ribosomal protein S6. Although further studies are obviously needed to validate the anticancer effects of this agent, our present results suggest that hyaluromycin is a promising drug for the treatment of PDAC.

HA is known to be present in a variety of molecular weights in pathological conditions and the size of HA is important in terms of its effects on cancer progression. In particular, LMW-HA, rather than HMW-HA, is more likely to promote cancer growth and metastasis [8]. We also demonstrated that direct addition of LMW-HA to cultured PDAC cells increased their migration more robustly than HMW-HA [9]. Because HA is degraded by HYALs, accelerated HYAL activity is associated with aggressive tumor progression [3]. For example, overexpression of HYAL1, one of the major HYALs, results in increased cell proliferation, migration, invasion, and metastasis in prostate and breast cancer [14, 15].

Based on these findings, enhanced HYAL activity is considered an ideal target for cancer therapy. In fact, previous studies showed antitumor effects of several HYAL inhibitors. For example, glycyrrhizin, a HYAL inhibitor also known as an anti-inflammatory drug, inhibits the growth, migration, and metastasis of various cancers, including glioblastoma, lung cancer, and leukemia [10]. In addition, sulfated HA reported as an inhibitor of testicular HYALs more than 60 years ago [16] inhibits tumor growth mainly by inducing apoptosis in prostate cancer cells [11]. Furthermore, dextran sulfate, an inhibitor of HYAL [17], reduced migration and invasion of breast cancer cells by decreasing the endogenous LMW-HA levels [8]. We also demonstrated that inhibition of HYAL activity by dextran sulfate significantly inhibits the migration of PDAC cells [5]. Despite its role as an HYAL inhibitor, dextran sulfate was also known as a potent carcinogenic agent which has been used in an inflammation-related mouse colon carcinogenesis model [18, 19]. Our results suggest, for the first time, the antitumor effects of hyaluromycin in PDAC cells in associated with a decreased level of LMW-HA. Because of its origin from natural marine species and potent inhibitory activity against HYAL, hyaluromycin could be a promising antineoplastic drug for PDAC and, possibly, other cancers.

We also found that treatment with hyaluromycin resulted in decreased phosphorylation of ribosomal protein S6, a mediator of PI3K/Akt/mTOR pathway [20]. Similarly, other HYAL inhibitors are reported to target this pathway. For example, previous study showed that glycyrrhizin inactivated Akt/mTOR/STAT3 pathway [10]. Furthermore, sulfated HA inhibited PI3K/Akt pathway as the major HA signaling target [11]. Interestingly, overexpression of rpS6 and phosphorylation of rpS6 promoted the proliferation, migration, and invasion in non-small cell lung cancer cells [21]. Thus, inactivation of PI3K/Akt/mTOR pathway could be a possible mechanism underlying the antitumor effects of HYAL inhibitors. However, our study showed decreased phosphorylation of ribosomal protein S6 but not of Akt and mTOR after treating PDAC cells with hyaluromycin. It is possible that hyaluromycin could target phosphorylation of ribosomal protein S6 independent of the PI3K/Akt/mTOR pathway, but further investigation is definitely required to confirm our present results in future studies.

## 5. Conclusion

In summary, we demonstrate that hyaluromycin inhibits proliferation and migration of PDAC cells, at least in part, through decreased level of LMW-HA.

## Abbreviations

HA: Hyaluronan
PDAC: Pancreatic ductal adenocarcinoma
HYALs: Hyaluronidases
HMW: High-molecular-weight
LMW: Low-molecular-weight
sHA: Sulfated hyaluronic acid
FBS: Fetal bovine serum.
